# The network nature of language endangerment hotspots

**DOI:** 10.1038/s41598-022-14479-1

**Published:** 2022-06-24

**Authors:** Nala H. Lee, Cynthia S. Q. Siew, Nadine H. N. Ng

**Affiliations:** 1 Department of English, Linguistics and Theatre Studies, Faculty of Arts and Social Sciences, National University of Singapore, Block AS5, 7 Arts Link, Singapore, 117570 Singapore; 2 Department of Psychology, Faculty of Arts and Social Sciences, National University of Singapore, Block AS4, 9 Arts Link, Singapore, 117570 Singapore

**Keywords:** Biodiversity, Sustainability

## Abstract

Language endangerment is one of the most urgent issues of the twenty-first century. Languages are disappearing at unprecedented rates, with dire consequences that affect speaker communities, scientific community and humanity. There is impetus for understanding the nature of language endangerment, and we investigate where language endangerment occurs by performing network analysis on 3423 languages at various levels of risk. Macro-level analysis shows evidence of positive assortative mixing of endangerment statuses—critically endangered languages are surrounded by similarly endangered languages, indicating the prevalence of linguistic hotspots throughout the world. Meso-level analysis using community detection returned 13 communities experiencing different levels of threat. Micro-level analysis of closeness centrality shows that more geographically isolated languages tend to be more critically endangered. Even after accounting for the statistical contributions of linguistic diversity, the structural properties of the spatial network were still significantly associated with endangerment outcomes. Findings support that the notion of hotspots is useful when accounting for language endangerment but go beyond that to establish that quantifying spatial structure is crucial. Language preservation in these hotspots and understanding why endangered languages pattern the way they do in their environments becomes more vital than ever.

## Introduction

Language endangerment is recognized to be one of the most pressing contemporary issues. Languages have been observed to go extinct in the past and the phenomenon is viewed as being not uncommon, especially from a broad historical perspective^1^. Yet, in recent times, language endangerment is recognized to be taking place at an unprecedented rate and magnitude^2^. Different estimates have been provided: a more extreme prediction is that 50% of the world’s languages are endangered and that as many as 90% of the world’s languages can become moribund or extinct in this current century^3^. A less extreme but still highly consequential claim based on calculations made using recent data indicates that one language is lost every 3 months^4^. Regardless of the actual figure, the consequences of language loss are well documented as they are immense^5,6^. The outcomes of language loss include losses to the impacted language communities, to the scientific community, and overall, to humanity. Language loss is linked to the loss of cultural or ethnic identity^7^, and has been shown to affect the psychological well-being of affected speakers^8^ as well as bring about worse physical health outcomes in indigenous populations^9^. In addition, language loss constitutes the loss of cultural and linguistic diversity^10^, the loss of knowledge of prehistory by losing the only means of reconstructing a culture’s past^5^, and the loss of language data which compromises the abilities of linguists to understand the full extent of what the human brain and cognition are capable of^11^. All arguments provided here point to the immediate impetus of more comprehensively understanding the nature of language endangerment in order to respond more effectively to the issue at hand. Advancements have made data on endangered languages much more readily available than before and provided us with new computational tools that allow us to shed new perspective on the issue of language endangerment. Here, we are concerned with where language endangerment occurs, and we explore the nature of these locales with a data-driven approach.

Several uses of this research can be envisioned. The results of this study may inform language documentation and revitalization programs of language endangerment trends and illuminate what is at stake of being lost where. In terms of language endangerment itself, the research can form a basis for exploring the environmental commonalities between language hotspots that contain more languages that are at higher levels of risk than others, as well as differences between hotspots of differing natures. In terms of language documentation, such work can help prioritize where language documentation efforts should be first focused, given limited resources in terms of research personnel and funding^12^. Where language revitalization is concerned, work such as this can hopefully heighten the awareness of language endangerment among community members who live in particularly threatened hotspots. These are but some suggestions on how the work can be used.

Earlier studies on language hotspots have categorized languages by pre-demarcated regions and subsumed issues of language endangerment together with those of linguistic diversity and understudied languages^13^. The same broadness is applied in biocultural diversity, which overlays concepts and regions of biodiversity onto cultural and linguistic diversity, with an emphasis on the importance of carrying out the conservation of biodiversity together with that of language and culture^14–17^. However, there is work that suggests the importance of decoupling these concepts^18^. In that vein, we find there to be value in understanding the fundamental nature of language endangerment hotspots from the outset using quantitative methods. We then move on to make observations about the nature of linguistic diversity within these quantitatively motivated hotspots of language endangerment, which more fully emphasizes what is at risk of being lost where.

We performed a spatial network analysis on 3423 languages in the Endangered Languages Catalogue, which is hosted on the Endangered Languages Platform. The goal of the Catalogue is to present all languages that communities and scholars have pointed out to be at some level of risk, as well as languages that have become dormant within the last 50 years. In addition to being the largest database of endangered languages globally, the information in the Catalogue is constantly and periodically updated based on feedback gathered from language communities and scholars worldwide^19^. The data that is utilized for this project therefore represents what was most accurately known at its point of utilization. Crucially, the Catalogue also includes information regarding the level of endangerment each language is at and the language’s geographical coordinates. In our spatial network, each node represents the geographical “location” of an endangered language as indicated in the Catalogue, and the edges represent the Haversine distance between the longitude and latitude coordinates of the endangered languages (see “Methods” section). We leverage the tools of network science to analyze the structural characteristics of the endangered languages’ spatial network at its macro-level through an analysis of assortative mixing patterns, at its meso-level through community detection methods, and at its micro-level through an analysis of the languages’ closeness centralities (see “Methods” section).

## Main findings

Our analyses show that these endangered languages form natural clusters or hotspots geographically, and importantly, that these clusters occur and have meaningful implications for language endangerment *even after accounting for the level of linguistic diversity in those areas.*

At the *macro-level* of the network, there is evidence of positive assortative mixing by endangerment statuses, *r* = 0.21, *p* < 0.001. This result implies that languages that are critically endangered tend to be surrounded by other languages that are similarly critically endangered. Conversely, the opposite can also be said. Languages that are lower on the scale of endangerment tend to be surrounded by other languages that are similarly less endangered. The positive assortative mixing of endangerment status indicates the prevalence of language endangerment hotspots throughout the world. Essentially, this finding foregrounds the logical assumption that languages that cluster spatially will share environmental, social, economic, and historical influences^20^, or even experience similar policies, power relations, and patterns of migration.

**Figure 1:**
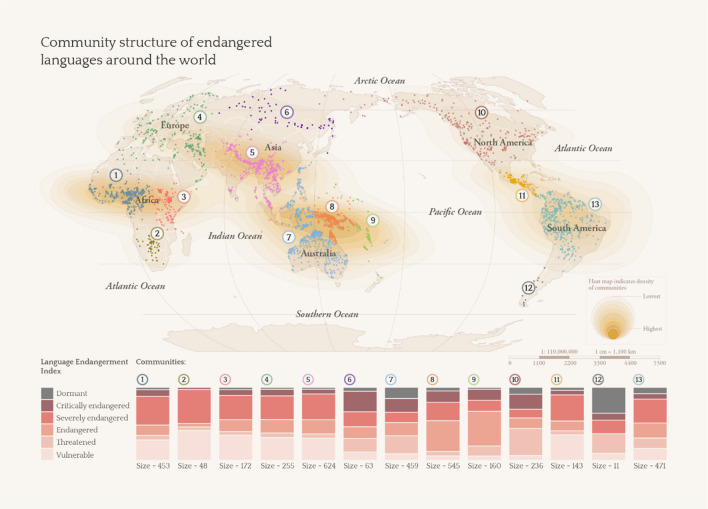
Community structure of endangered languages around the world.

Community detection analysis aims to uncover communities of nodes at the *meso-level* of the network. Here, community as a network science term refers to a subset of nodes that are more interconnected within that subset than outside of that subset. The Louvain community detection method was applied to our network and returned 13 communities ranging in size from 11 to 624 languages with an overall modularity value of 0.77, indicating that there is robust community structure in the network. These communities are found in regions that correspond to (1) West Africa; (2) Southern Africa; (3) East Africa; (4) Western Europe, Middle East, and Northern Africa; (5) Central Asia (southern), East Asia (southern), South Asia, and mainland Southeast Asia; (6) Eastern Europe, Central Asia (northern) and East Asia (northern); (7) Island Southeast Asia and Australia; (8) Melanesia; (9) Micronesia, Polynesia and New Zealand; (10) Northern America and parts of Northern Asia; (11) Central America; (12) South America (southern); and (13) South America (northern). A qualitative examination of these communities further reveals that some communities fare worse than others in terms of having proportionally more languages at higher levels of endangerment than others. The communities that have more dormant and critically endangered languages include those of (12) South America (southern), (7) Island Southeast Asia and Australia, (10) North America and parts of Northern Asia, as well as (6) Eastern Europe, Central Asia (northern) and East Asia (northern) (see Fig. 1).

Our findings reflect an observation made in the early 1990s that North American languages and Australian languages have been affected tremendously by endangerment, but disagree with the observation that the languages within Central America are faring better^3^. Our findings, however, accord with a qualitative account from the 2000s that states that all the indigenous languages in the region are considered to be endangered^21^. Our findings are also partially similar to more recent research that states that languages of the Americas and the Pacific are most at risk^14^, as well as research that situates the top five language hotspots in the Northwest Pacific Plateau, Central South America, Central Siberia, Eastern Siberia, and Northern Australia^13^. Note that while some of these communities appear to be meaningful in terms of language families, such as community (8) which reflects the endangered Austronesian and Papuan languages of Melanesia, and (9) which reflects the endangered Austronesian languages of Remote Oceania, other communities reflect disparate groupings, such as community (7), which includes the Austronesian and Papuan languages of Island Southeast Asia, as well as the Pama-Nyungan languages of Australia. Rather, these communities are best construed as geographic connectivity patterns that occur due to the spatial patterning of languages on the globe, given the nature of spatial network analysis.

Another question that immediately arises involves the nature of hotspots that are threatened. There may be multiple factors that are at play at each hotspot, plausibly including state formation decimating linguistic diversity in (5) Central Asia (southern), East Asia (southern), South Asia, and mainland Southeast Asia and (6) Eastern Europe, Central Asia (northern) and East Asia (northern), with political complexity being associated with the spread of ethnolinguistic groups^22^, or even a lack of direct resources for language maintenance^20^ in regions such as (8) Melanesia. Here, we are interested in why certain hotspots are more critically endangered than others. Recent research covering languages globally suggests that multiple variables can drive language loss, including greater road density as well as higher average years of schooling, and importantly, that direct contact with neighboring languages in itself is not a threatening process^20^—in line with our findings that the most threatened hotspot is found in the (12) South America (south) region, where languages are highly isolated geographically. Notably, the highly isolated languages found here have extremely small speaker numbers, which reflects the state of endangerment of these languages. The three languages that are southernmost on the tip of South America are Yagan, Kawésqar, and Tehuelche. Yagan—also spelled Yahgan, Yaghan, and Yagán and known as Yamana, Yámana, Yamaná, Yapoo, Tequenica, and Háusi Kúta—is a language isolate that is unrelated to other languages; it is now only spoken by one elderly female speaker who remains in Villa Ukika on Isla Navarino in Chile^23^. Kawésqar, which is also spelled Kawesqar, Kaweskar, Qawasqar, Kawashkar, Qawashqar and known as Halakwalup, Pecheré, and Alacaluf, is also found in Chile. It is the only remaining representative of its small language family and is spoken by about ten speakers^24^. Tehuelche—also spelled Tewelche and known as Aoniken, Aonek'enk, Aonek'enk, Inaquen, Inaquean, Chon, Tsoneka, Gununa-Kena, and Gününa Küna—is a Chonan language spoken by nomadic hunters who once could be found in present-day Chile. The language is said to be spoken by only three speakers in Argentina today^25^ (see Catalogue for more information).

With the spatial nature of our data and analysis, and the qualitative observation that the most critically endangered hotspot appears to be characterized by highly isolated languages, we explore the spatial nature of each endangered language. We then use a *micro-level* network measure known as closeness centrality to quantify the relative position (or distance) of each endangered language to all other languages in the network. Specifically, languages with high closeness centralities tend to be “centrally” located in the network whereas languages with low closeness centralities tend to be located on the periphery of the network. A 1-way between-groups ANOVA indicated that languages found at the peripheries of the network are more likely to be more critically endangered than those at the core of the network, *F*(5, 3634) = 12.43, *p* < 0.001. Post-hoc analyses further indicate that the more geographically isolated a language is, the more likely it is to be critically endangered. These findings explain why the most threatened hotspots are more threatened than others—they contain more geographically isolated languages. The ways in which geographical isolation affects the endangerment statuses of languages can and should be explored beyond the scope of this paper, but again, a lack of direct resources for language maintenance^20^, as well as rural-to-urban migration patterns^26,27^ may affect the continuity of the languages concerned.

In our final analysis, we wished to investigate if the relationship between the spatial characteristics of endangered languages (as operationalized by the micro-level network measure of closeness centrality) and their endangerment statuses would still hold even after taking into account the level of linguistic diversity in the region. This is an important question to ask, given the assumed overlaps between the level of language endangerment and the level of linguistic diversity. A much-asserted consequence of language loss is also the loss of linguistic diversity^10,28^, which presumes that these two parameters must be somewhat related. There exists work on language hotspots that has treated levels of language endangerment and linguistic diversity (as well as level of language documentation) as logically independent parameters that nevertheless manifest in concentrations of diverse, endangered (and poorly documented) languages around the globe^13^. Recent work on endangerment and linguistic diversity observes that areas with the greatest absolute number of endangered languages also coincide with regions containing the most diversity, possibly because there are simply more languages in these regions to be endangered^20^. Our findings show however, that the correlation between levels of endangerment and linguistic diversity is less straightforward, given that our identified communities differ in size, and it is the smallest community that appears to be most threatened—i.e. (12) South America (southern) region, thus motivating the need to explore the role of linguistic diversity. In our analysis, linguistic diversity is operationalized as the number of (unique) language families and isolates in each of the 13 communities returned by the community detection analysis above.

Ordinal regression analysis revealed that both the closeness centrality network measure and linguistic diversity were significant predictors of endangerment status. Specifically, languages that are found in more central locations in the network (i.e., higher closeness centrality, odds ratio = 0.94, z = − 1.98, p < 0.05) and in less linguistically diverse regions (lower diversity coefficient, odd ratio = 1.33, z = 9.67, p < 0.001) tend to be associated with better outcomes. Conversely, languages that are found in peripheral locations in the network and in more linguistically diverse regions tend to be associated with worse outcomes.

While language endangerment and linguistic diversity have been either presumed to be related or regarded as independent parameters, our study represents an important attempt to operationalize and quantitatively assess how these two constructs are associated with the spatial structure of endangered languages. Although language endangerment and linguistic diversity are positively correlated (r = 0.71, p < 0.001), our analysis shows that even after accounting for the statistical contributions of linguistic diversity, the structural properties of the spatial network were still significantly associated with endangerment outcomes.

Overall, our study demonstrates a number of important results. First, assortativity analysis indicates that linguistic hotspots are prevalent throughout the world. Second, community detection analysis identifies specific language communities in which there are more languages at a higher level of risk than others. Third, languages that are spatially isolated tend to be more critically endangered. Finally, linguistic diversity of the region that endangered languages are found in cannot fully account for their endangerment levels; the spatial structures of where endangered languages are found must also be considered. Together, the findings from our study support that the notion of hotspots is useful when accounting for language endangerment^13–15^ but go beyond that to suggest that quantifying the spatial structure of endangered languages is crucial. Situating language endangerment research within the broader spatial context can help researchers understand the environmental mechanisms that may drive language endangerment. In effect, the findings of this study can aid triage efforts where language documentation and revitalization are concerned. Work on language documentation and revitalization in the hotspots that we have identified is vital, particularly along the peripheries of the network where languages experience higher levels of endangerment. Equally crucial will be research that can uncover why endangered languages pattern the way they do in the geographical environments that they are found in.

## Methods

### Database utilized

The database comprises information obtained with permission from the Catalogue of Endangered Languages that is hosted on the Endangered Languages Project platform (https://www.endangeredlanguages.com/). The Endangered Languages Project was first developed and launched by Google, and is currently overseen by First People’s Cultural Council and the Institute for Language Information and Technology at Eastern Michigan University. Information about the languages in this project is provided by the Catalogue, which is produced by the University of Hawai’i at Mānoa and Eastern Michigan University, with funding provided by the U.S. National Science Foundation (Grants #1058096 and #1057725) and the Luce Foundation. The project is supported by a team of global experts comprising its Governance Council and Advisory Committee.

In general, the Catalogue aims to present all languages that communities and scholars have pointed out to be at some level of risk as well as languages that have become dormant. In addition to being the largest database of endangered languages globally, the Catalogue is updated periodically based on feedback gathered from language communities and scholars worldwide. The data therefore represents what was most accurately known about the state of each language’s vitality at its point of utilization. At the time of usage, there were 3423 languages represented in the Catalogue that were determined to be at various levels of risk. Assessment of each language's risk level is carried out using the Language Endangerment Index, which was developed for the Catalogue’s purposes. The Index is used to assess the level of endangerment of any given language based on whether there is intergenerational transmission of the language (whether the language is being passed on to younger generations), its absolute number of speakers, speaker number trends (whether numbers are stable, increasing, or decreasing), and domains of language use (whether the language is used in a wide number of domains or limited ones). The levels of endangerment that the Index generates include ‘safe’, ‘vulnerable’, ‘threatened’, ‘endangered’, ‘severely endangered’, and ‘critically endangered’. Languages for which it remains unclear if the language has gone extinct or whose last fluent speaker is reported to have died in recent times are referred to as ‘dormant’. Given that the focus of the Catalogue is languages that are at some level of threat, safe languages are excluded in general. Where locality information is available, each language is also accompanied with its latitudinal and longitudinal coordinates.

#### Steps taken to prepare the data for network analysis

The data obtained from the Catalogue was further organized and cleaned up for analysis.

1. Identifier code
  Where available, the ISO 639-3 code for each language was utilized as its unique identifier. Otherwise, its LINGUIST List local use code was utilized. These are temporary codes that are not in the current version of the ISO 639-3 Standard for languages. For languages with neither, unique 3-letter codes were constructed.
2. Endangerment level
  Each language’s endangerment level appeared together with a level of certainty score in the same cell in the original data file. Both pieces of information were split into separate columns and only endangerment levels were utilized.
  For languages where different data were available in the Catalogue depending on resource utilized, the data was listed in additional columns. The endangerment level data points utilized in these cases were the ones with the most complete and updated information. If there was no data available regarding endangerment level, this information was also reflected.
3. Coordinates
  Where exact coordinates were not available, coordinates were approximated using Google maps based on the location description provided in the Catalogue source (e.g., the Tel Aviv district), attained from other sources such as Glottolog, UNESCO Atlas of the World’s Languages in Danger, or approximated from maps provided in other sources. ‘NA’ was indicated in the field for coordinates if none could be found.
  Coordinates found to be inaccurate were rejected, for example in the instance that coordinates provided indicate a different location than the country the language is supposedly found in. The above steps were then taken to populate the coordinates field.
  In instances where a language appears in more than one country, these are listed in separate rows as separate entries. Where there are two sets of coordinates for a country, the set that best corresponds with the written description in the Catalogue source, has greater detail, or is more recent is chosen. Where there are more than two sets of coordinates, a middle point is chosen as being representative of the language’s location, by plotting all coordinates on MapCustomizer (www.mapcustomizer.com).
4. Language family
  On the Catalogue, the information regarding language family may be multi-tiered. For example, Laghuu falls under the Lolo-Burmese branch of the Sino-Tibetan family. For this study, the broader family is utilized—in the case of Laghuu the label ‘Sino-Tibetan’ is used.
  Mixed languages, pidgins, and creoles have all been categorized as ‘contact languages’.
  Language isolates are listed as ‘isolates’.
5. Region

The Catalogue groups ‘Mexico, Central America, Caribbean’ together under region. Central America and Caribbean are listed as separate regions in this study, with Mexico falling under Central America.

### Network construction

A spatial network of endangered languages was constructed from the database. Each node represented an endangered language, and edges or links depicted the distance between the locations of the languages as specified in the database. A distance matrix containing the distances between all endangered languages was computed by using functions from the ‘geosphere’ R package. Specifically, Haversine distances were computed for each pair of longitude and latitude points in the dataset. The radius of the earth used in the Haversine distance calculation is 6,378,137 m (for more details see: https://www.rdocumentation.org/packages/geosphere/versions/1.5-14/topics/distHaversine). Haversine distance refers to the shortest distance between two points on a spherical earth, also referred to as the "great-circle-distance"^29^.

#### Sensitivity analyses of edge thresholds

The distance matrix is a fully connected network with weighted, undirected links. We set out to capture the strongest or "closest" spatial relationships among the endangered languages, therefore an edge threshold was applied to the distance matrix such that only the edges in the **x**th lowest percentile were retained in the spatial network. Such an approach allows for the analysis of the most meaningful (i.e., the physically closest) spatial relations in the dataset and how they relate to language endangerment status. The edges were then transformed into unweighted connections to create a simple unweighted, undirected graph for analysis. In order to determine the value of x (i.e., the percentile at which the edge threshold is to be applied), we constructed 10 spatial networks that retained edges with distances below the 1st, 2nd, 3rd… 10th percentile (in increments of 1%) of all distances in the matrix. Additional information of the distances depicted by the edges in each of the 10 networks is provided in Supplementary Information.

These 10 networks were then analyzed for their macro- and meso-scale network properties. A summary of macro and meso-scale network measures used in this analysis and their definitions is provided in Table 1, which depicts the 10 networks showing similar patterns in their network structures.

**Table 1:** An overview of macro- and meso-level network measures of spatial networks with different thresholds.

Measure		Threshold									
		1	2	3	4	5	6	7	8	9	10
**V**	Number of nodes	3883	3883	3883	3883	3883	3883	3883	3883	3883	3883
**E**	Number of edges	75,369	150,737	226,104	301,473	376,842	452,210	527,578	602,946	678,315	753,683
Network density	Proportion of observed edges/number of possible edges (corresponds to threshold, **x**)	0.01	0.02	0.03	0.04	0.05	0.06	0.07	0.08	0.09	0.10
Average degree, **k**	Average number of connections or edges each node has	38.8	77.6	116.5	155.3	194.1	232.9	271.7	310.6	349.4	388.2
Global clustering coefficient, **C**	Measure of local clustering (closed triangles)	0.796	0.821	0.843	0.83	0.819	0.815	0.815	0.819	0.822	0.822
Average shortest path length, **ASPL**	Average length of the shortest path between all possible node pairs in the network	9.71	11.04	14.83	10.46	12.94	10.75	9.13	7.97	7.15	6.36
Components	Number of distinct connected components	213	60	28	17	5	4	3	3	3	3
lcc_prop_	Proportion of nodes in the largest connected component of the network	0.249	0.463	0.756	0.757	0.996	0.996	0.999	0.999	0.999	0.999
Modularity, **Q**	Meso-level metric quantifying the robustness of community structure (subclusters) of the network	0.79	0.79	0.79	0.79	0.77	0.75	0.73	0.71	0.71	0.7

#### Results

As expected, network density and average degree of the networks, which serve as indicators of the number of edges relative to the number of nodes in the network, increased as the edge threshold used to connect nodes became more liberal. The relatively high values of C (i.e., high levels of local clustering among nodes) and low values of ASPL (i.e., relatively short paths despite large size of network) suggested the presence of small world structure^30^. The community detection analysis using the Louvain method^31^ indicated strong evidence of community structure in the networks—suggesting the presence of clusters of endangered languages.

The point at which the vast majority of nodes was located within the largest connected component of the network occurred at the 5% edge threshold. Because the 5% network was not too fragmented, we report the analyses conducted on the largest connected component of the 5% network in the following subsections. Please see Supplementary Information for additional details behind the rationale for selecting the 5% network for further analyses. The smaller connected components were excluded. Note however that our results are robust across spatial networks of various edge thresholds (due to lack of space, please see Supplementary Information for a complete summary of all reported analyses conducted on all 10 spatial networks).

### Macro-level analysis: assortative mixing of endangerment statuses

#### Method

To investigate the macro-level structure of the spatial network of endangered languages, we computed the assortativity coefficient of the spatial network. Specifically, we wanted to know if the endangerment statuses of the languages tended to cluster at the global level of the entire network. If the assortativity coefficient is positive, the languages in the network would tend to be connected to languages of similar levels of endangerment. If the assortativity coefficient is negative, the languages in the network would tend to be connected to languages of dissimilar levels of endangerment.

#### Results

**Figure 2:**
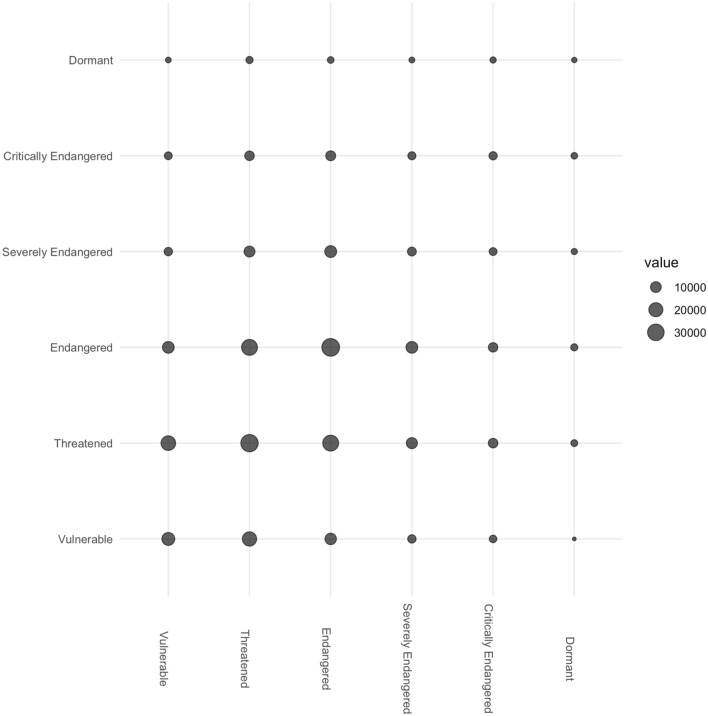
Bubble plot of endangerment statuses among spatially close languages.

There is a significant positive correlation (Spearman's rank correlation) between the endangerment status of connected pairs of endangered languages in the network, r = 0.20, p < 0.001. This indicates that languages that are more endangered tend to be connected to (hence, close to) languages that are also more endangered. Figure 2 shows a bubble plot of endangerment statuses among spatially close languages: The larger bubbles toward the diagonal as compared to the edges of the plot indicate the presence of positive assortative mixing patterns in the network.

### Meso-level analysis

Many real-world networks from diverse domains have robust community structure^32^. Broadly speaking, communities are defined as groups of nodes in the network that are more interconnected with each other than with nodes outside of the community^33^. Networks with robust community structure will have high values of modularity, Q, a network science measure that quantifies the density of connections within and across communities^33^.

Here, we applied the community detection method to our spatial network of endangered languages to investigate the following question: Are there particular language communities that show more severe endangerment levels? In other words, do more endangered languages tend to cluster around specific communities or are they found across all communities in the network?

1. Do data-driven approaches such as community detection return "meaningful" communities (groups of endangered languages) that correspond or align with regions identified in previous work?
2. Are there particular language communities that show more severe endangerment levels? In other words, do more endangered languages tend to cluster around specific communities or are they found across all communities in the network?

#### Method

We applied a community detection algorithm to the largest connected component of the 5% network. The largest connected component (LCC) is the network component containing the largest number of nodes that are connected to each other in a single component.

Although many community detection methods exist, we used the Louvain method^31^ as it is an efficient method that works well for large graphs. The general idea behind this approach is to reassign nodes to communities such that the highest contribution to modularity can be achieved. The reassignment process stops when the modularity of the network cannot be improved further. Specifics of the method can be found in Blondel et al.^31^.

#### Results

**Figure 3:**
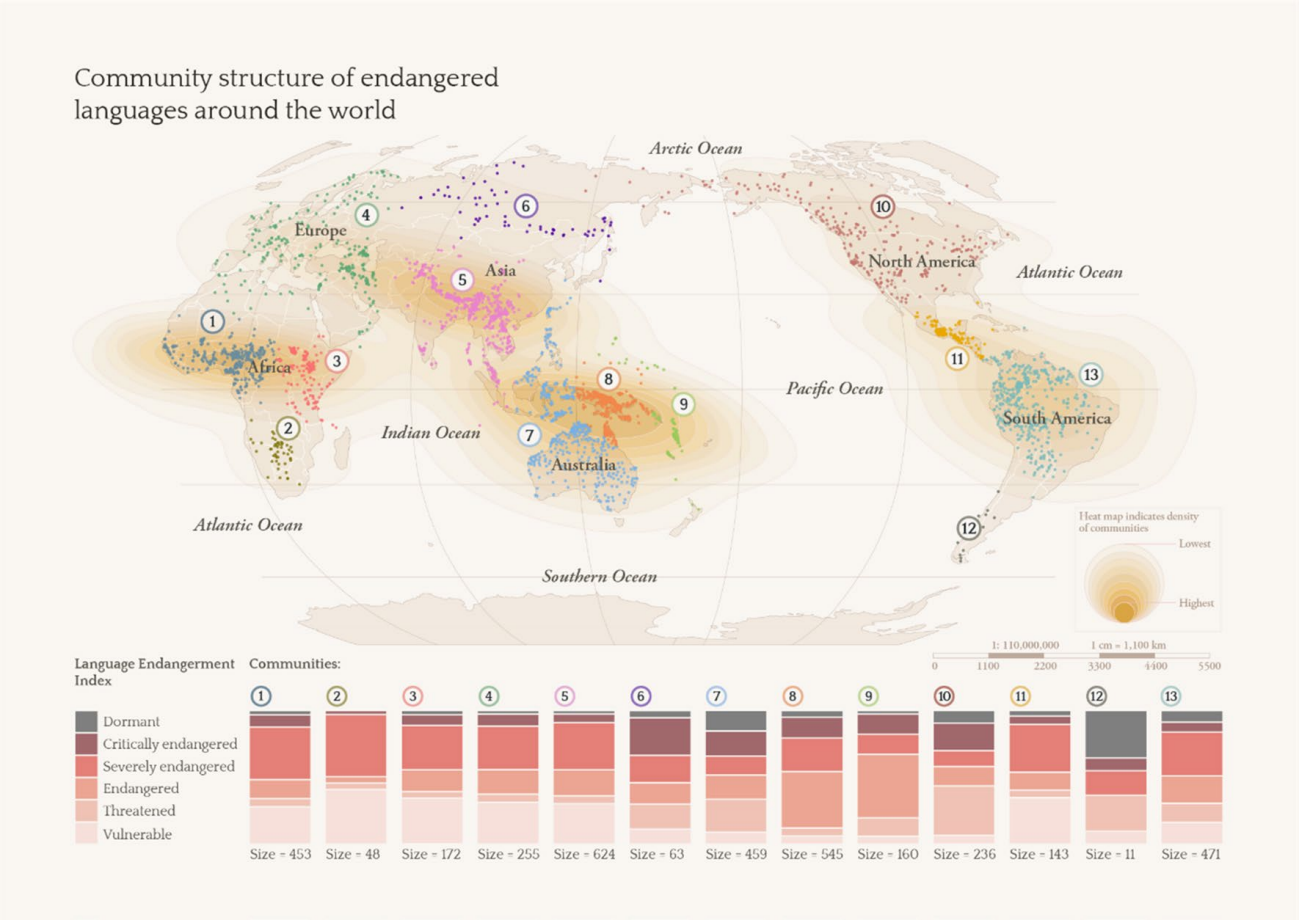
Community structure of endangered languages around the world.

The community detection method returned 13 communities, ranging in size from 11 to 624. Modularity, Q, was 0.77, indicating high levels of community structure of the network. Figure 1 of the findings section (replicated here as Fig. 3) shows the 13 communities and their properties.

Qualitatively, we observe that these communities correspond well to known or existing language regions. We also observe that certain communities have a much higher proportion of languages that are especially endangered (i.e., with larger darker stacks: Communities 12, 7, 10, 6). A more detailed discussion is provided in “Main findings” section.

### Micro-level analysis: the protective value of high closeness centralities

#### Method

Closeness centrality is a network science measure that measures the "centrality" of nodes in the network. Mathematically, it is the mean of the shortest paths between a target node and all other nodes in the network. Hence, it provides a way to measure a node’s importance by considering its distance in relation to other nodes in the network (see Table 2).

**Table 2:** Means and standard deviations of closeness centrality across languages of different endangerment statuses.

Level	Count	Mean	SD	Median
Vulnerable	715	0.083	0.021	0.079
Threatened	1070	0.082	0.020	0.080
Endangered	825	0.084	0.018	0.086
Severely endangered	417	0.083	0.016	0.086
Critically endangered	426	0.078	0.017	0.076
Dormant	202	0.075	0.016	0.079

#### ANOVA results

Overall, 1-way ANOVA comparing the closeness centralities of endangered languages across their statuses was statistically significant, *F*(5, 3634) = 12.47, *p* < 0.001. Post-hoc multiple comparisons (Tukey's test, with corrected family-wise p-values) were conducted. Overall, this analysis revealed that more endangered languages have lower closeness centralities. In other words, highly endangered languages tend to lie on the periphery of the network whereas languages that are less endangered tend to be found in the center of the spatial network (i.e., more centrally located in the network).

### The relationship between linguistic diversity, spatial network structure, and endangerment status

#### Method

##### Language families and isolates

A parent language and all its derived daughter languages are one unique representation of each language family. A language isolate does not belong to a wider family but can be considered to be a unique representation of itself, therefore constituting its own family. Our paper considers each unique representation in its count of linguistic diversity. Contact languages are left out of the count of these counts, their classification being unclear.

1. Operationalization of language families and isolates: based on pre-defined regions in the database
  - The languages were grouped by ‘region’ as defined in the Catalogue.
  - The number of unique linguistic families per region and isolates were counted. Items within the categories of ‘contact language’, ‘unclassified’ and ‘sign language’ were excluded from the count. This value is entered into the regression analysis below.
2. Operationalization of language families and isolates: based on community detection results
  - The languages are grouped by ‘community’ based on the output of the community analysis.
  - The number of unique linguistic families per community and isolates were counted. Items within the categories of ‘contact language’, ‘unclassified’ and ‘sign language’ were excluded from the count. This value is entered into the regression analysis below.

We note that these two measures of language families and isolates are highly positively correlated, r = + 0.71, p < 0.001.

#### Results

Model performance indices (see Table 3) indicate that the model containing the diversity (language families and isolates) coefficient derived from communities of nodes in the spatial network is a better model than the one containing the diversity coefficient derived from pre-defined regions in the Catalogue.

**Table 3:** Model performance indices for regression models containing language families and isolates derived from pre-defined regions (top) and from groups found in the community detection analysis (bottom).

Model	AIC	BIC	R2
Pre-defined	12,127.35	12,170.75	0.033
Data-driven	12,081.18	12,124.57	0.045

As seen in Table 4, the odds ratio of closeness centrality is less than 1, indicating that greater closeness centralities are associated with lower probabilities of a more severe language endangerment status. Languages that are more centrally positioned have less severe language endangerment statuses. The odds ratio of diversity is positive, indicating that greater linguistic diversity in the region is associated with higher probabilities of a more severe language endangerment status.

**Table 4:** Odds ratios for predictors in the model containing language families and isolates (community-based) and closeness centrality.

Predictors	Endangerment level		
	Odds ratios	*z*	*p*
Closeness centrality	0.94	− 2.06	0.040
Diversity	1.33	9.63	< 0.001
Observations	3640		
R^2^ Nagelkerke	0.045		

## Supplementary Information

Supplementary Information.

## Data Availability

The Catalogue of Endangered Languages dataset is publicly available for download and use from the Endangered Languages Project portal (https://endangeredlanguages.com/userquery/), under the terms and conditions of the Creative Commons Attribution 4.0 Unported (CC BY 4.0) license. Codes utilized and additional information regarding languages with coordinate issues have been made available here: https://github.com/csqsiew/language-endangerment.

## References

[1] Swadesh M (1948). Sociological notes on obsolescent languages. Int. J. Am. Linguist..

[2] Campbell L (2017). On how and why languages become endangered: Reply to Mufwene. Language.

[3] Krauss M (1992). The world’s languages in crisis. Language.

[4] Campbell, L. & Okura, E. New knowledge produced by the Catalogue of Endangered Languages. In *Cataloguing the World’s Endangered Languages* (eds. Campbell, L. & Belew, A.) 79–84. (Routledge, 2018).

[5] Evans N (2010). Dying Words: Endangered Languages and What They Have to Tell Us.

[6] Nettle D, Romaine S (2000). Vanishing Voices: The Extinction of the World’s Languages.

[7] Tsunoda T (2005). Language Endangerment and Language Revitalization: An Introduction.

[8] Hallett D, Chandler MJ, Lalonde CE (2007). Aboriginal language knowledge and youth suicide. Cogn. Dev..

[9] Flood D, Rohloff P (2018). Indigenous languages and global health. Lancet Glob. Health.

[10] Hale K (1992). Language endangerment and the human value of linguistic diversity. Language.

[11] Lee NH, Van Way J (2016). Assessing levels of endangerment in the Catalogue of Endangered Languages (ELCat) using the Language Endangerment Index (LEI). Lang. Soc..

[12] Hauk, B. & Heaton, R. Triage: Setting priorities for endangered language research. In *Cataloguing the World’s Endangered Languages* (eds. Campbell, L. & Belew, A.) 259–304 (Routledge, 2018).

[13] Anderson GDS (2011). Language hotspots: What (applied) linguistics and education should do about language endangerment in the twenty-first century. Lang. Educ..

[14] Loh J, Harmon D (2014). Biocultural Diversity: Threatened Species, Endangered Languages.

[15] Gorenflo LJ, Romaine S, Mittermeier RA, Walker-Painemilla K (2012). Co-occurence of linguistic and biological diversity in biodiversity hotspots and high biodiversity wilderness areas. Proc. Natl. Acad. Sci. U. S. A..

[16] Maffi L (2005). Linguistic, cultural, and biological diversity. Annu. Rev. Anthropol..

[17] Harmon, D., Loh, J., Congruence between species and language diversity. In *The Oxford Handbook of Endangered Languages* (eds. Rehg, K. & Campbell, L.) 659–682 (Oxford University Press, 2018).

[18] Turvey ST, Pettorelli N (2014). Spatial congruence in language and species richness but not threat in the world’s top linguistic hotspot. Proc. R. Soc. B.

[19] *Cataloguing the World’s Endangered Languages*. (Routledge, 2018).

[20] Bronham L, Dinnage R, Skirgård H (2022). Global predictors of language endangerment and the future of linguistic diversity. Nat. Ecol. Evol..

[21] Grinevald, C. Endangered languages of Mexico and Central America. In *Language Diversity Endangered* (ed. Brenzinger, M.) 59–86 (Mouton de Gruyter, 2006).

[22] Currie TE, Mace R (2009). Political complexity predicts the spread of ethnolinguistic groups. Proc. Natl. Acad. Sci. U.S.A..

[23] Crevels, M. Language endangerment in South America: The clock is ticking. In *The Indigenous Languages of South America: A Comprehensive Guide* (eds. Campbell, L. & Grondona, V.) 167–233 (Mouton de Gruyter, 2012).

[24] Becerra R (2019). Kawésqar (Chile)—Language Snapshot. Lang. Doc. Descr..

[25] Crevels, M. South America. In *Atlas of the World’s Endangered Languages* (ed. Moseley, C.) 103–196 (Routledge, 2007).

[26] Zhao Y (1999). Leaving the countryside: Rural-to-urban migration decisions in China. Am. Econ. Rev..

[27] Grau HR, Aide M (2008). Globalization and land use transitions in Latin America. Ecol. Soc..

[28] Crystal D (2000). Language Death.

[29] Sinnott RW (1984). Virtues of the haversine. Sky Telesc..

[30] Watts DJ, Strogatz SH (1998). Collective dynamics of ‘small-world’ networks. Nature.

[31] Blondel VD, Guillaume JL, Lambiotte R, Lefebvre E (2008). Fast unfolding of communities in large networks. J. Stat. Mech..

[32] Fortunato S (2010). Community detection in graphs. Phys. Rep..

[33] Newman ME (2006). Modularity and community structure in networks. Proc. Natl. Acad. Sci. U. S. A..

