# Supplementary material for: The network nature of language endangerment hotspots
Source: Sci Rep. 2022 Jun 24;12:10803. doi: 10.1038/s41598-022-14479-1 (PMC9232642; doi:10.1038/s41598-022-14479-1)
Supplement: Supplementary file 1 — Supplementary Information. [file 41598_2022_14479_MOESM1_ESM.docx]

Supplementary Information for "The network nature of language endangerment hotspots"

# Selection process of edge threshold

Figure 1 demonstrates how the number of network components decreases as the edge threshold becomes more liberal, where nodes are connected if their distances are less than the value necessary to "fill in" 1% through 10% of all possible edges in the network. Figure 1 shows quantitatively the level of fragmentation across the thresholds. Based on a visual inspection, our rationale for using the 5% threshold was that this appeared to be the point at which almost all nodes were in the largest connected cluster, and it is also the point at which an increasingly liberal threshold leads to decreasing marginal gains in the reduction of the number of components in the network.

*Figure 1. A scree plot showing the number of distinct network components declining as the edge threshold used in the network construction increases.*


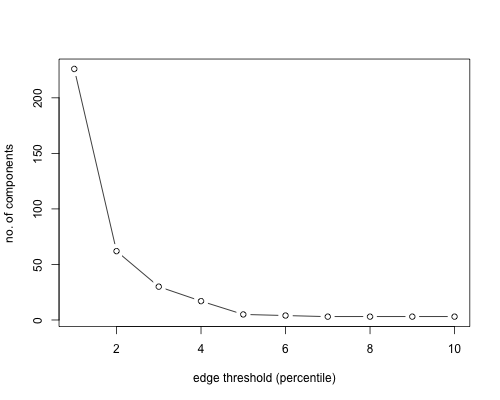


# Sensitivity analyses demonstrating robustness of results across different edge thresholds

In this section, we report the results of all analyses conducted in the main manuscript across 10 networks constructed with differing edge thresholds.

## Analysis 1: Assortative mixing (macro-level analysis)

Across all 10 networks, there is evidence of positive assortative mixing of language endangerment statuses, all *p*s < .001 (see Table 1).

*Table 1. Spearman’s rank correlations of the endangerment status of connected pairs of endangered languages in the network.*

| Edge threshold | rho | p-value |
| --- | --- | --- |
| 1 | 0.26 | < .001 |
| 2 | 0.24 | < .001 |
| 3 | 0.22 | < .001 |
| 4 | 0.21 | < .001 |
| 5 | 0.20 | < .001 |
| 6 | 0.20 | < .001 |
| 7 | 0.19 | < .001 |
| 8 | 0.18 | < .001 |
| 9 | 0.18 | < .001 |
| 10 | 0.18 | < .001 |

## Analysis 2: Community analysis (meso-level analysis)

We also implemented the Louvain algorithm to detect communities in each of the 10 networks (see Table 2). We observe more stable results and generally higher modularity scores for networks constructed with thresholds of 3^rd^ percentile and upwards. Notably, the 5% network had the highest modularity score of 0.77, indicating highly robust sub-clusters of nodes in the 5% network.

*Table 2. Summary of community detection results.*

| Edge threshold |  | Number of communities | Smallest community size | Largest community size | Modularity, Q |
| --- | --- | --- | --- | --- | --- |
| 1 |  | 13 | 18 | 185 | 0.41 |
| 2 |  | 11 | 42 | 392 | 0.61 |
| 3 |  | 16 | 18 | 453 | 0.74 |
| 4 |  | 15 | 19 | 500 | 0.73 |
| 5 |  | 13 | 11 | 660 | 0.77 |
| 6 |  | 12 | 52 | 662 | 0.75 |
| 7 |  | 11 | 11 | 697 | 0.73 |
| 8 |  | 8 | 52 | 1031 | 0.71 |
| 9 |  | 7 | 162 | 990 | 0.71 |
| 10 |  | 7 | 161 | 991 | 0.70 |

## Analysis 3: Closeness centrality (micro-level analysis)

Tables 3 to 12 demonstrate the ANOVA results for each of the 10 networks. The general result reported in the main manuscript held across the different edge thresholds - languages found at the peripheries of the network are more likely to be more critically endangered than those at the core of the network.

*Table 3. ANOVA results for 1% network.*

| threshold 1: *F*(5,922) = 31.97, *p* < .001 | | | | |
| --- | --- | --- | --- | --- |
| level | **count** | **mean** | **sd** | **median** |
| Vulnerable | 715 | 0.103 | 0.042 | 0.101 |
| Threatened | 1070 | 0.120 | 0.034 | 0.141 |
| Endangered | 825 | 0.128 | 0.028 | 0.142 |
| Severely Endangered | 417 | 0.112 | 0.029 | 0.111 |
| Critically Endangered | 426 | 0.095 | 0.027 | 0.093 |
| Dormant | 202 | 0.095 | 0.028 | 0.092 |

*Table 4. ANOVA results for 2% network.*

| threshold 2: *F*(5,1715) = 21.09, *p* < .001 | | | | |
| --- | --- | --- | --- | --- |
| level | **count** | **mean** | **sd** | **median** |
| Vulnerable | 715 | 0.080 | 0.018 | 0.074 |
| Threatened | 1070 | 0.086 | 0.020 | 0.081 |
| Endangered | 825 | 0.092 | 0.018 | 0.102 |
| Severely Endangered | 417 | 0.093 | 0.017 | 0.102 |
| Critically Endangered | 426 | 0.090 | 0.017 | 0.092 |
| Dormant | 202 | 0.091 | 0.014 | 0.093 |

*Table 5. ANOVA results for 3% network.*

| threshold 3: *F*(5,2764) = 2.66, *p* = .02 | | | | |
| --- | --- | --- | --- | --- |
| level | **count** | **mean** | **sd** | **median** |
| Vulnerable | 715 | 0.066 | 0.016 | 0.071 |
| Threatened | 1070 | 0.067 | 0.015 | 0.071 |
| Endangered | 825 | 0.069 | 0.012 | 0.071 |
| Severely Endangered | 417 | 0.067 | 0.011 | 0.070 |
| Critically Endangered | 426 | 0.067 | 0.011 | 0.069 |
| Dormant | 202 | 0.065 | 0.008 | 0.065 |

*Table 6. ANOVA results for 4% network.*

| threshold 4: *F*(5,2770) = 2.43, *p* = .03 | | | | |
| --- | --- | --- | --- | --- |
| level | **count** | **mean** | **sd** | **median** |
| Vulnerable | 715 | 0.094 | 0.021 | 0.098 |
| Threatened | 1070 | 0.095 | 0.019 | 0.099 |
| Endangered | 825 | 0.097 | 0.016 | 0.099 |
| Severely Endangered | 417 | 0.094 | 0.015 | 0.098 |
| Critically Endangered | 426 | 0.094 | 0.015 | 0.094 |
| Dormant | 202 | 0.092 | 0.011 | 0.091 |

*Table 7. ANOVA results for 5% network.*

| threshold 5: *F*(5,3634) = 12.47, *p* < .001 | | | | |
| --- | --- | --- | --- | --- |
| level | **count** | **mean** | **sd** | **median** |
| Vulnerable | 715 | 0.083 | 0.021 | 0.079 |
| Threatened | 1070 | 0.082 | 0.020 | 0.080 |
| Endangered | 825 | 0.084 | 0.018 | 0.086 |
| Severely Endangered | 417 | 0.083 | 0.016 | 0.086 |
| Critically Endangered | 426 | 0.078 | 0.017 | 0.076 |
| Dormant | 202 | 0.075 | 0.016 | 0.079 |

Table 8. ANOVA results for 6% network.

| threshold 6: *F*(5,3634) = 11.34, *p* < .001 | | | | |
| --- | --- | --- | --- | --- |
| level | **count** | **mean** | **sd** | **median** |
| Vulnerable | 715 | 0.099 | 0.024 | 0.095 |
| Threatened | 1070 | 0.098 | 0.023 | 0.097 |
| Endangered | 825 | 0.101 | 0.021 | 0.109 |
| Severely Endangered | 417 | 0.100 | 0.019 | 0.106 |
| Critically Endangered | 426 | 0.094 | 0.021 | 0.094 |
| Dormant | 202 | 0.091 | 0.020 | 0.096 |

*Table 9. ANOVA results for 7% network.*

| threshold 7: *F*(5,3646) = 11.66, *p* < .001 | | | | |
| --- | --- | --- | --- | --- |
| level | **count** | **mean** | **sd** | **median** |
| Vulnerable | 715 | 0.117 | 0.027 | 0.112 |
| Threatened | 1070 | 0.115 | 0.026 | 0.115 |
| Endangered | 825 | 0.118 | 0.023 | 0.125 |
| Severely Endangered | 417 | 0.117 | 0.021 | 0.121 |
| Critically Endangered | 426 | 0.110 | 0.023 | 0.110 |
| Dormant | 202 | 0.107 | 0.021 | 0.111 |

*Table 10. ANOVA results for 8% network.*

| threshold 8: *F*(5,3646) = 10.19, *p* < .001 | | | | |
| --- | --- | --- | --- | --- |
| level | **count** | **mean** | **sd** | **median** |
| Vulnerable | 715 | 0.134 | 0.030 | 0.130 |
| Threatened | 1070 | 0.132 | 0.029 | 0.130 |
| Endangered | 825 | 0.134 | 0.026 | 0.143 |
| Severely Endangered | 417 | 0.134 | 0.024 | 0.142 |
| Critically Endangered | 426 | 0.127 | 0.026 | 0.127 |
| Dormant | 202 | 0.122 | 0.025 | 0.129 |

*Table 11. ANOVA results for 9% network.*

| threshold 9: *F*(5,3646) = 11.26, *p* < .001 | | | | |
| --- | --- | --- | --- | --- |
| level | **count** | **mean** | **sd** | **median** |
| Vulnerable | 715 | 0.149 | 0.033 | 0.143 |
| Threatened | 1070 | 0.147 | 0.033 | 0.146 |
| Endangered | 825 | 0.151 | 0.030 | 0.163 |
| Severely Endangered | 417 | 0.150 | 0.027 | 0.156 |
| Critically Endangered | 426 | 0.141 | 0.029 | 0.140 |
| Dormant | 202 | 0.136 | 0.028 | 0.140 |

*Table 12. ANOVA results for 10% network.*

| threshold 10: *F*(5,3646) = 10.66, *p* < .001 | | | | |
| --- | --- | --- | --- | --- |
| level | **count** | **mean** | **sd** | **median** |
| Vulnerable | 715 | 0.166 | 0.035 | 0.162 |
| Threatened | 1070 | 0.164 | 0.035 | 0.165 |
| Endangered | 825 | 0.169 | 0.032 | 0.177 |
| Severely Endangered | 417 | 0.168 | 0.029 | 0.177 |
| Critically Endangered | 426 | 0.159 | 0.031 | 0.159 |
| Dormant | 202 | 0.154 | 0.030 | 0.159 |

## Analysis 4: Controlling for the influence of linguistic diversity

Table 13 provides summary of the odds ratios for closeness centrality and linguistic diversity coefficients in the ordinal regression analyses at each of the 10 networks. We observe that the results become stable for networks constructed with thresholds of 3^rd^ percentile and upwards, whereby the odds ratio of closeness centrality is < 1, indicating that languages that are found in more central locations in the network tend to be associated with better outcomes, and the odds ratio of linguistic diversity is > 1, indicating that less linguistically diverse regions tend to be associated with better outcomes. It is important to note that in the less liberal thresholds of 1^st^ and 2^nd^ percentile, there were much fewer observations due to high fragmentation and a lack of nodes in the largest connected component which led to the inability of computing closeness centrality of nodes for a large proportion of the data.

*Table 13. Summary of odds ratios for predictors in the ordinal regression models.*

| Edge threshold | Closeness centrality | Linguistic diversity | Number of observations |
| --- | --- | --- | --- |
| 1 | 0.96 | 0.54 | 928 |
| 2 | 1.96 | 0.64 | 1721 |
| 3 | 0.98 | 1.30 | 2606 |
| 4 | 0.90 | 1.29 | 2710 |
| 5 | 0.94 | 1.33 | 3640 |
| 6 | 0.99 | 1.34 | 3640 |
| 7 | 0.97 | 1.33 | 3652 |
| 8 | 0.96 | 1.63 | 3652 |
| 9 | 0.98 | 1.60 | 3652 |
| 10 | 0.97 | 1.58 | 3652 |

# Interpretation of edge thresholds

Table 14 provides additional descriptive details about the length of the edges for all 10 networks. In particular, the maximum distance (in kilometers) provides some intuition about the "furthest" two locations that would be connected at each threshold.

*Table 14. Descriptive statistics of the distances depicted by the edges in each network.*

| Edge threshold | Number of edges | Mean (km) | SD | Median (km) | Max (km) |
| --- | --- | --- | --- | --- | --- |
| 1 | 75369 | 163.26 | 78.42 | 165.07 | 294.29 |
| 2 | 150737 | 288.10 | 144.65 | 294.29 | 528.00 |
| 3 | 226104 | 404.42 | 205.72 | 413.40 | 746.68 |
| 4 | 301473 | 518.05 | 267.43 | 528.00 | 969.73 |
| 5 | 376842 | 628.79 | 327.02 | 636.33 | 1171.44 |
| 6 | 452210 | 735.52 | 382.90 | 746.68 | 1368.12 |
| 7 | 527578 | 839.63 | 437.18 | 859.65 | 1557.95 |
| 8 | 602946 | 941.06 | 489.50 | 969.73 | 1744.78 |
| 9 | 678315 | 1040.88 | 541.34 | 1072.12 | 1935.25 |
| 10 | 753683 | 1139.85 | 593.46 | 1171.44 | 2126.43 |
